# Palliative sedation for children at end of life: a retrospective cohort study

**DOI:** 10.1186/s12904-022-00947-y

**Published:** 2022-04-27

**Authors:** Yang Chen, Jianjun Jiang, Wei Peng, Chuan Zhang

**Affiliations:** 1 Department of Palliative Medicine, West China School of Public Health and West China Fourth Hospital, Sichuan University, No. 18, Section 3, South Renmin Road, Wuhou District, Chengdu, Sichuan province China; 2 Department of Palliative Medicine, West China School of Public Health and West China Fourth Hospital, Sichuan University; West China – PUMC C.C. Chen Institute of Health, Sichuan University, No. 18, Section 3, South Renmin Road, Wuhou District, Chengdu, Sichuan province China

**Keywords:** Palliative sedation, Children, Palliative care, Symptom control, End of life

## Abstract

**Background:**

Palliative sedation is consciously reducing the patient’s consciousness to alleviate the refractory symptoms. However, studies on palliative sedation for children are scarce. We aimed to survey the symptom control and risks for children with sedative therapy in end of life.

**Method:**

This study was a single center retrospective cohort study. Children who died in the Department of Palliative Medicine were divided into palliative sedation (Group A) and non-palliative sedation group (Group B). The symptoms relief, survival time, and last hospitalization time were compared between two groups.

**Results:**

From January 2012 to November 2019, 41 children died in department of palliative care. 24 children were sedated (Group A), meanwhile 17 children were not (Group B). The symptoms in Group A were more complex than Group B (*p* = 0.013). Overall symptom relief in Group A was higher than that in Group B (24/24, 10/15 *p* = 0.041). Pain relief rates (7/7, 20/21 *p* = 0.714), maximum/pre-death opioid dose [30(20, 77.5), 18(9, 45) *p* = 0.175, 30(20, 60), 18(9, 45) *p* = 0.208] and pain intensity difference [5(4,6.5), 4(2,6) *p* = 0.315] did not differ significantly in either groups. After diagnosis, the survival time of the Group A was longer than the Group B (*p* = 0.047). However, the length of hospitalization before death was similar in two groups (*p* = 0.385).

**Conclusion:**

Palliative sedation controls complicated, painful symptoms at the end of life and does not shorten the hospitalization time in children.

## Background

The annual demand for palliative care for children in China ranges from 23 to 24 per 100,000 people [1], with approximately 3,037,950 cases of children in need of pediatric palliative care among 1.3 billion people [2]. In 2017, the Integrated National Mortality Surveillance System, which covers 24% of China’s population, showed that 90,600 deceased children may have benefited from palliative care [3].

Palliative care not only improves the physical and psychosocial symptoms of children with limited lives [4, 5] but also benefits the children themselves and their families [6–10]. There is now an increasing social emphasis on timely intervention in palliative care for children. Such care could control symptoms, reduce overtreatment and allow for end of life preparation of those children. These factors contribute to improving psychosocial outcomes during parental bereavement [11–13]. Various symptoms appear at the end of a child’s life, which are intractable, unmanageable and unrelievable and cause great suffering and distress to children, their families and caregivers [14, 15]. Palliative sedation, which aims to relieve end of life refractory symptoms by reducing patient consciousness, was reported to be used for 12–64% of adults in palliative care [16–18] and for 48.4% of adults who die in hospitals at end of life with refractory symptoms [19]. Two studies in mainland China revealed that 22.3–33.6% of adult patients who died in the Palliative Department or Integrated Therapy Department received some form of palliative sedation [20, 21]. Although there has been a demonstrated benefit of better symptom control for patients with a terminal illness, palliative sedation remains somewhat controversial [22, 23].

In the available studies, palliative sedation in children has been reported as isolated cases and shared experiences [24–27], but there is a lack of efficacy and safety evidence for palliative sedation in children. At present, there are no reports of palliative sedation for children in China. The aim of this study was to describe the sedation procedure, to assess the means for symptom control and the risks for children at the end of life to enrich the practical experience for palliative sedation in children.

## Methods

### Patients

We retrospectively recorded pediatric patients admitted to the palliative care department of our hospital between January 2012 and November 2019. The inclusion criteria were as follows: 1. age of 0–18 years; 2. patients who received palliative care; and 3. patients who died during hospitalization. The exclusion criteria were 1. age > 18 years; 2. no death during hospitalization; and 3. incomplete information.

### Methods

#### Groups

Children were divided into a palliative sedation group (Group A) and a nonpalliative sedation group (Group B) according to whether they were administered sedative drugs (midazolam, chlorpromazine), and the demographic characteristics, principal diagnosis, types of symptoms at admission and hydration during hospitalization of the two groups were compared.

##### Observation

Information about symptom relief, pain, dyspnea, survival time after diagnosis, and last hospitalization time was extracted from the electronic medical records. Symptoms mentioned in the medical records, such as pain, dyspnea, agitation, coma, convulsions, vomiting, and fever, were recorded. Symptom control was divided into no remission (none of the symptoms were relieved), partial remission (greater than or equal to one symptom was relieved) and complete remission (relief of all symptoms).

The pain parameters were pain intensity difference (PID), pain relief rate, and pain relief (PAR) [28]. Pain intensity (PI) assessments used the Face, Legs, Activity, Cry, Consolability (FLACC) scale, Wang-Baker faces pain rating scales, or numerical rating scale (NRS), according to age and cognitive ability. Analgesic drugs (initial dose, maximum dose, predeath dose and route), pain relief, and length of sleep were recorded. The dose of opioids every 24 h was converted to oral morphine (conversion rate, oral morphine: intravenous morphine =3:1, oral morphine: subcutaneous morphine =2:1, oral morphine: fentanyl transdermal patch (25 mcg/h) = 60:25, and oral morphine: oral oxycodone =2:1) [29–31]. The pain intensity difference (PID) indicated the difference between the pain score at the beginning of the administration of analgesics and the pain score at each time point. Pain relief (PAR) was graded as no remission, mild remission (pain relief of approximately 1/4), moderate remission (pain relief of approximately 1/2), significant remission (pain relief of more than 3/4).

##### Dyspnea

Breathing, shortness of breath, gasping, or dyspnea mentioned in the medical records were considered dyspnea. Dyspnea relief included 1. clearly documented alleviation after treatment and 2. if symptoms were not mentioned later, symptoms were deemed to be relieved.

##### Statistical analysis

Statistical analyses were performed using SPSS 20.0. Descriptive statistics (e.g., mean, SD, median, interquartile range) were used for analysis, and frequencies were calculated for continuous and categorical data. For comparisons of continuous data/ordinal data and categorical data, we used the Wilcoxon rank sum test and the chi-square test. Spearman’s rank correlation was used for the correlation test. *P* values lower than 0.05 were considered indicative of statistical significance.

##### Ethics

The study was approved by the medical ethics committee of West China Fourth Hospital of Sichuan University (No. HXSY-EC-2021027).

## Results

### Demographic characteristics of groups A and B

Between January 2012 and November 2019, 80 children were admitted to the Department of Palliative Medicine, West China Fourth Hospital, and 41 of them died during their hospitalization. There were 24 patients in the palliative sedation group (Group A) and 17 in the nonpalliative sedation group (Group B). The demographic characteristics of the two groups are shown in Table 1; differences between the two groups were not statistically significant.

**Table 1:** Demographic characteristics of the palliative sedation and nonpalliative sedation groups

Demographic characteristics (*N* = 41)	Group A (*n* = 24)	Group B (*n* = 17)	*p*
**Age**			
0–28 days	0	3	0.228^&^
29 days −1 year	3	1	
2–6 years	11	6	
7–18 years	10	7	
**Sex**			
Male	15	13	0.40^&^
Female	9	4	
**Type of disease**			
Blood tumor	4	3	0.564^&^
Solid tumor	16	8	
Congenital disease	2	3	
Diseases of the blood system	0	1	
Severe infection	2	2	
**Hydration during hospitalization**	21	14	0.679^&^

^&^Fisher’s exact probability method

### Symptoms

**Fig. 1:**
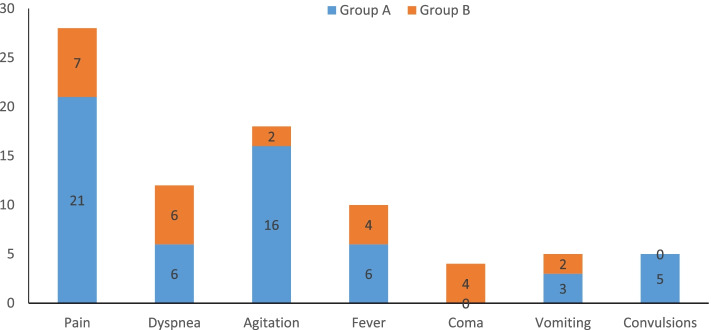
The distribution of the main symptoms in Groups A and B

The main symptoms were pain, dyspnea, agitation, fever, coma, vomiting, and convulsions (see Fig. 1). Pain, which occurred in 28/41 (68%) children, was the most common symptom affecting the children’s quality of survival at the end of life. Four children in group A had four types of symptoms, ten children had three types, seven children had two types, and three children had one symptom. In group B, none of the children had four symptoms, four children had three symptoms, three had two symptoms, and ten had one symptom. The distribution of symptoms in the two groups is shown in Table 2. Types of symptoms differed between the two groups, *p* = 0.013. The symptoms in group A were more complex than those in group B. However, overall symptom relief was higher in Group A, which received sedative medication (*p* = 0.041). The unrelieved symptoms of five patients in Group B were fever, abdominal distension, and dyspnea. Three of the five children did not have a tumor. The pain control rate was similar between the two groups, 95.23 and 100%.

**Table 2:** Types and relief of symptoms in Groups A and B

	n	Type of symptom (s)					Symptom control			Pain control				
		1	2	3	4	n	No remission	Partial remission	Complete remission	n	No remission	Mild remission	Moderate remissions	Obvious remission
Group A	24	3	7	10	4	24	0	20	4	21^b^	1^c^	2	16	2
Group B	17	10	3	4	0	15^a^	5	8	2	7^b^	0	2	4	1
Z		–					2.045				0.367			
*p*	0.013^d^						0.041^e^				0.714 ^e^			

^a^Two cases of only coma excluded from the 17 cases were not included in the assessment

^b^There was no analgesia for 3 patients in Group A and 10 patients in Group B

^c^Hospital stay was too short to evaluate

^d^The exact probability method

^e^Rank sum test

### Pain control

Twenty-one children in group A used analgesics, and three patients in group A were not prescribed analgesia because their main symptoms were convulsions or abdominal distension accompanied by moaning. Seven children in group B used analgesics (7/17 patients who had fever or coma were not prescribed analgesics, and 3/17 patients who were prescribed morphine only because of dyspnea were not included in the pain assessment).

Twenty-one children in group A and seven in group B had been prescribed opioid analgesics. Pain scores on admission and after control were higher in group A than in group B (*p* = 0.014, 0.039), but there was no significant difference in maximum opioid dosage and predeath opioid dosage between the two groups. Pain control is shown in Table 3.

**Table 3:** Pain control in Groups A and B

	N	Admission			After symptom control			Maximum opioid dosage converted to oral morphine dosage/24 h (mg)	Opioid dosage before death converted to oral morphine dosage/24 h (mg)	Pain intensity difference
		Pain score	Pain duration (hours)	Sleep (hours)	Pain score	Pain duration (hours)	Sleep (hours)			
Group A	21	8(7,10)	24 (20,24)	6 (4,7)	3 (3,4)	4 (2.5,7)	8 (6,10.5)	30 (20,77.5)	30 (20,60)	5 (4,6.5)
Group B	7	7 (6,7)	22 (18,24)	4 (4,5.5)	2 (0,3)	4 (1.5,13)	9 (8)	18 (9,45)	18 (9,45)	4 (2,6)
Z		−2.501	−0.095	−1.481	−2.068	− 0.065	0.615	− 1.358	−1.278	− 1.004
*p*		0.014	0.924	0.139	0.039	0.948	0.538	0.175	0.208	0.315

Opioids were the first choice for moderate and severe pain in children at the end of life. In our study, before hospital admission, 24 children were treated with morphine sulfate/hydrochloride; 2, with oxycodone hydrochloride; 1, with fentanyl; and 1, with an acetaminophen and hydrocodone mixture. The forms included sustained-release tablets, oral liquid, injection, and transdermal patches. Two children were treated with two types of opioids (morphine sulfate oral solution + morphine hydrochloride injection, fentanyl transdermal patch + morphine sulfate oral solution). Sixteen patients used only single opioid analgesia, and 12 patients used 1–3 adjuvant analgesic drugs, including paracetamol, ketorolac trometamol, scopolamine butyrate, gabapentin capsule, valproate sodium, and ketamine.

### Dyspnea

In the study, twelve children, aged 4 days to 14 years, had dyspnea. Three patients had dyspnea as a single symptom, and the other nine had pain, agitation, fever, and other symptoms. There were six patients in Group A and six patients in Group B. Both groups had 83% remission of dyspnea, and both groups used morphine to relieve dyspnea. The survival time and hospitalization time of the two groups are shown in Table 4 (the data are nonnormal and expressed as quartile spacing). The sample size of children with dyspnea was too small to analyze the data.

**Table 4:** Relief rate and survival time of dyspnea patients in Groups A and B

	n	Dyspnea relief rate %(n)	Survival time after diagnosis of disease^a^ (days)	Length of hospitalization^a^ (days)
Group A	6	83%(5)	450 (253.5,1036.5)	7.5 (1,21.5)
Group B	6	83%(5)	63.5 (52.5,1855)	12 (1.75,51.75)

^a^Quartile spacing

### Duration of survival and hospitalization time after diagnosis in group A and group B

The survival time after diagnosis in Group A was 20–2190 days, and the hospitalization time was 1–85 days. The duration of survival in Group B was 5–5475 days, and the length of hospital stay was 1–63 days (see Table 5). A longer survival time after disease diagnosis was observed in Group A than in Group B (*p* = 0.047). However, the hospitalization time before death was similar. Palliative sedation treatment did not seem to shorten the hospitalization time of children at the end of life.

**Table 5:** Length of survival after diagnosis and length of hospitalization before death in Groups A and B

	Length of survival after diagnosis of disease	Previous length of hospitalization
Group A	365 (112.5,730)	9 (2.5,22.75)
Group B	60 (30,795)	3 (1,24)
*Z*	−1.987	−0.869
*p*	0.047	0.385

### Sedation in group A

In electronic records, children (age, from 5 months to 14 years; 15 males; 4 blood tumors, 16 solid tumors, 2 congenital diseases, 2 infections) were prescribed sedative drugs. There were between one and four main symptom types (pain, dyspnea, agitation, fever, coma, vomiting, convulsions). Palliative sedation was mainly administered due to agitation in 13 patients, pain in 8 patients, dyspnea in 6 patients and convulsions in 5 patients. Midazolam was used in 17 patients; chlorpromazine was used in 1 patient. Six patients used midazolam combined with chlorpromazine. The hospital stays lasted from 1 h to 85 days, and the use of sedatives lasted from 1 h to 47 days. The purpose of sedation was to reduce consciousness to control painful symptoms. Before and after sedation, the average Ramsay scores were 1 (1, 1) and 2 (2, 3), respectively. When death occurred, a deeper sedation score of 3 (2, 4) was observed (*p* < 0.01) (see Table 6). At the initiation of sedation, the initial dosage of midazolam ranged between 0.42 and 16.67 μg/kg.h [median 3.21 (1.81–6.51) μg/kg.h]. The maximum dosage was 0.42–65.1 μg/kg.h [median 8.77 (1.81–23.81) μg/kg.h], and the predeath dosage was 0.41–32 μg/kg.h [median 8.77 (1.81–16.67) μg/kg.h]. The maximum dosage of midazolam was similar to that before death (*p* = 0.068). For 4 patients, the midazolam dosage was reduced before death for unknown reasons, but the degree of sedation was not reduced. The midazolam sedation duration did not appear to be associated with the maximum dosage or predeath dosage.

**Table 6:** Ramsay scores of the sedation group

	Ramsay score ^a^
Presedation score	1 (1,1)
Sedation score after symptom control	2 (2,3)
Predeath sedation score	3 (2,4)
*X*^2^	34.344
*P* ^*b*^	< 0.01

^a^Percentile spacing

^b^Friedman inspection

## Discussion

Children experience refractory symptoms at the end of life. These symptoms seriously affect the quality of life of the children and their caregivers and place a heavy mental and psychological burden on medical workers [14, 15]. Palliative sedation consists of purposefully reducing a patient’s consciousness to alleviate symptoms. From January 2012 to November 2019, 58.5% (24/41) of children who died in the palliative care department were treated with palliative sedation, similar to the palliative sedation rate reported in previous studies [27, 32–35]. However, a report from Spain revealed that 12.2% (20/164) of children received palliative sedation at the end of life [36]. The discrepancy in the sedation rate might be partly attributable to the lack of a unified definition and lack of any clear indication of palliative sedation. Alternatively, there were differences between the respective study cohorts regarding clinical and cultural settings.

End of life symptoms in children are complex, and pain is the most common symptom that affects children’s quality of life [37, 38], followed by dyspnea, agitation fever, and other symptoms. Symptom complexity in the palliative sedation group was higher than that in the nonsedation group. In our study the idications for sedation were agitation, pain, dyspnea and convulsion, most child had more than one symptoms.

When a child was admitted to the palliative department, the physician assessed the patient’s symptoms and provided routine treatment to correct reversible factors. Once conventional interventions failed, or if the child was intolerant to current treatment, the symptoms were deemed refractory. Palliative sedation could then be discussed between palliative specialists and the family. Light sedation was initiated. Sedatives were titrated until symptoms were relieved.

During palliative sedation management, midazolam was started at a low dosage and titrated until the refractory symptoms were relieved. The midazolam maximum dosage varied greatly between individuals, and the average Ramsay score after symptom control was 2 (percentile spacing 2–3). Most patients were sedated to scores of 2–3, and only two children had a Ramsay score of 5. All sedation continued until death, and the sedation duration ranged from 1 h to 47 days. There was no difference in hospitalization duration between the sedation group and the nonsedation group. Sedation until death should not shorten the hospitalization time of children. During sedation, the children were all properly rehydrated, and there was no serious damage due to rehydration. Is it necessary to reduce the sedative dose to change the level of sedation when patients have reached deep sedation? For four children, the midazolam dosage was reduced before death, but the level of sedation did not improve.

Opioids were the first choice for moderate and severe pain in children at the end of life [39]. In our study, before hospital admission, 24 children were treated with morphine sulfate/hydrochloride; 2, with oxycodone hydrochloride; 1, with fentanyl; and 1, with an acetaminophen and hydrocodone mixture. The forms included sustained-release tablets, oral liquid, injection, and transdermal patches. Two children were treated with two types of opioids (morphine sulfate oral solution + morphine hydrochloride intravenous infusion, fentanyl transdermal patch + morphine sulfate oral solution). Sixteen patients used only single opioids, and 12 patients used 1–-3 adjuvant analgesic drugs, including paracetamol, ketorolac trometamol, scopolamine butyrate, gabapentin capsule, valproate sodium, and ketamine. The sedation group had higher pain scores at admission. The combination of sedative drugs and opioids avoided higher opioid doses.

End-of-life dyspnea was treated with routine treatment, including anti-infection drugs, bronchodilators, glucocorticoids, and other drugs, to correct reversible factors. All children with dyspnea used morphine to relieve dyspnea, and six children were prescribed palliative sedation. Both the sedation and nonsedation groups achieved a high rate of symptom relief (both 83%).

This is the first study to describe procedures addressing refractory symptoms for children at the end of life. This study found that palliative sedation could relieve refractory symptoms for children at the end of life but did not hasten death. However, the symptoms of the sedation group were more complex than those of the nonsedation group, and the symptom relief rate was higher in the sedation group. Titration with a sedative to alleviate suffering was a safe management strategy for children in the palliative department. Sedation until death should not shorten the end of life for children. The presence of multiple symptoms and refractory symptoms makes it easier for doctors and families to make decisions to opt for palliative sedation. Midazolam is a commonly used drug for palliative sedation in adults [40]. It is still the first choice for palliative sedation in children in our hospital because of its rapid effect and short duration of action. Severe adverse reactions, such as central inhibition and respiratory inhibition, were not observed after titration.

Notably, palliative sedation decisions may be associated with symptom complexity, the degree of pain, refractory dyspnea, the disease course, disease severity and expected survival time, as well as healthcare professionals’ perceptions of palliative sedation and parental wishes. All cases were discussed before sedation by a team of palliative medicine experts to determine that the symptoms were refractory, the disease progression was irreversible and the child’s survival time was limited. Subsequently, a family meeting was organized, and an informed consent form was signed. None of these children attended this meeting before sedation because they were not of legal age to be their own agents. All palliative sedation informed consent forms were signed by their guardians.

Palliative sedation for children seemed effective and safe in this study. Our study was a retrospective study and had a small sample size. There are fewer children in palliative care than there are adults or elderly patients. Therefore, multicenter clinical studies are needed for further confirmation.

Palliative sedation has been debated by society and the medical field. Whether to involve the child in the decision-making discussion about sedation and the use of sedation until death remains an ethical discussion.

## Conclusion

Palliative sedation controls complicated, painful symptoms at the end of life and does not shorten the hospitalization time in children.

## Data Availability

The data used and analyzed during the current study are available from the corresponding author upon reasonable request.
